# Simulation-free cone beam CT-based online adaptive radiotherapy for metastatic spinal cord compression

**DOI:** 10.2340/1651-226X.2025.44040

**Published:** 2025-08-25

**Authors:** Lisette Juul Sten, Evangelos Giannoulis, Laura Ann Rechner, Lina Åström, Anna Mann Nielsen, Jens Morgenthaler Edmund, Gitte Fredberg Persson

**Affiliations:** a Department of Oncology, Copenhagen University Hospital – Herlev and Gentofte, Herlev, Denmark; b Department of Clinical Medicine, University of Copenhagen, Copenhagen, Denmark

**Keywords:** Online adaptive radiotherapy, cone-beam computed tomography, simulation-free, ethos, metastatic spinal cord compression, HyperSight

## Abstract

**Background and purpose:**

A simulation-free approach, using the patient’s diagnostic computed tomography (CT) for treatment planning, eliminates the need for a separate planning CT. Combined with conebeam computed tomography (CBCT)-guided online adaptive radiotherapy (oART), this strategy has the potential to create a more efficient treatment workflow and reduce the burden for the patients.

The study aimed to evaluate the feasibility and time consumption of different simulation-free oART workflows for patients with metastatic spinal cord compression (MSCC) to identify the most suitable option for clinical implementation.

**Patient/material and methods:**

Diagnostic CT scans from patients diagnosed with MSCC were used for treatment planning, while CBCT scans from their first treatment session were retrospectively used to emulate the treatments. Four adaptive workflows were defined and assessed: Deformable Supervised (DefSup), Deformable Unsupervised (DefUn), Rigid Supervised (RigSup), and Rigid Unsupervised (RigUn). The supervised workflows involved manual corrections to the target structures, whereas the unsupervised workflows did not include any manual adjustments. Time stamps, segmentation quality, and dose plans were used to evaluate the feasibility of each workflow.

**Results:**

A total of 120 simulation-free emulations were performed (based on 27 patients with 30 target sites). The DefSup workflow yielded the highest accuracy in both segmentation and dose distribution. Additionally, with a median time consumption of 6.57 min, this workflow demonstrates a level of reliability and quality suitable for clinical application.

**Interpretation:**

The DefSup workflow was found to be the most optimal and safe for clinical implementation, as demonstrated by the successful treatment of the first patient with MSCC using this approach.

## Introduction

Metastatic spinal cord compression (MSCC) is a severe complication of metastatic cancer, affecting approximately 19% of patients with advanced-stage disease [1, 2]. MSCC requires urgent treatment within 1 or 2 days after diagnostic confirmation with whole-spine magnetic resonance imaging (MRI). Most patients are treated with palliative radiotherapy using a conventional workflow that necessitates an additional planning CT (pCT) scan for target delineation and treatment planning [3]. This process can be time-consuming and may require additional hospital visits, placing strain on an already frail and often highly symptomatic patient population. This burden is especially pronounced for ambulatory patients, who must travel from home for each appointment, thereby increasing their discomfort and the burden associated with the transportation [4].

Online adaptive radiotherapy (oART) allows for online dose plan re-optimization based on a cone beam computed tomography (CBCT) of the patient in the treatment position [5–8]. Recent studies have explored the feasibility of a simulation-free workflow, where a diagnostic CT (dCT) is used for treatment planning. With this workflow, the pCT can be omitted and the patient can meet directly for treatment delivery [9–12]. This approach, however, faces challenges related to CBCT image quality and dose calculation accuracy [13, 14]. Additionally, patient-related factors such as variations in positioning, anatomical changes, and prolonged time on the treatment couch, which can be uncomfortable or painful for patients, also pose limitations [12].

The primary objective of this study was to identify the optimal workflow for clinical implementation of simulation-free oART for patients with MSCC assessed by target dose coverage accuracy and time consumption.

## Patients/material and methods

### Study type and patient selection

Eligible patients for this retrospective simulation study were treated with non-adaptive image-guided radiotherapy (IGRT) for MSCC on a Halcyon or Ethos system (Varian, a Siemens Healthineers Company, Palo Alto, CA) between September 2023 and April 2024. All patients were treated according to the conventional workflow, which involved a pCT.

Exclusion criteria were spinal surgery after the dCT, cervical targets, and cases with extensive soft tissue involvement. All study participants had given consent in advance to use their data in clinical research.

### Data collection

A dCT and MRI were retrieved from the clinical imaging database (IMPAX) for all included patients and imported to the ARIA Treatment Planning System (TPS) (Varian, a Siemens Healthineers Company, Palo Alto, CA) for target delineation. Regarding the dCT, no specific criteria were set for the time interval between image acquisition and the first treatment. The only requirement for its use in a simulation-free approach was that the target area was clearly visualized.

The HyperSight CBCT from the patients’ first treatment session was retrieved from the Ethos TPS. All DICOM files were anonymized before being imported to the various TPS systems.

### Delineations

A radiation oncologist contoured the reference Clinical Target Volume (CTV) on the dCT guided by MRI. Contouring of the CTV was performed in accordance with the institution’s local delineation guidelines, which specify that the CTV should include only the involved vertebra(e), specifically the vertebral body, posterior arch, and spinal canal. The transverse and spinous processes are included only if disease involvement is confirmed by MRI.

A 5 mm margin was added to the CTV to create the Planning Target Volume (PTV). The department’s clinical constraints for targets and OARs are included in the Supplementary, supplementary materials, Table 1. The constraints define the proportion of the target volume that must receive a certain percentage of the prescribed dose. For instance, V97% ≥ 100% means that at least 97% of the target volume should receive 100% of the dose.

### Treatment planning

Based on the dCT, a reference plan was created in the Ethos emulator (v2.0) TPS. Clinical objectives established for targets and OARs were translated into optimization goals using the Intelligent Optimization Engine (IOE) in Ethos TPS [15]. A prescription of 25Gy in five fractions was chosen for emulation of the treatments. Only one fraction was emulated to balance pragmatic considerations, and a 7-field Intensity-Modulated Radiotherapy (IMRT) plan was selected for treatment planning.

### Adaptive workflows

**Figure 1:**
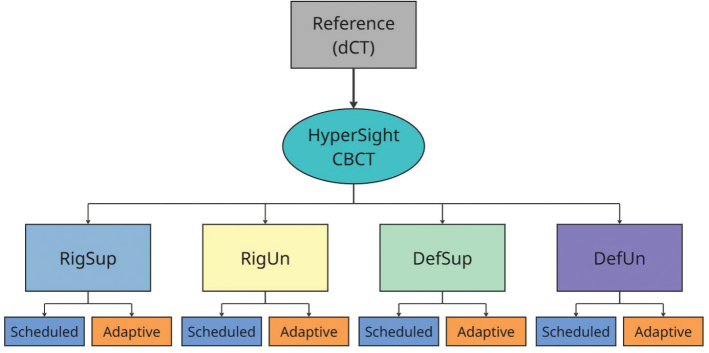
Overview of the collected imaging data and the subsequent workflows. The diagnostic CT (dCT) was used as the reference scan for contouring and treatment planning. The HyperSight CBCT scan was used for treatment simulation in the four defined workflows: Rigid Supervised (RigSup), Rigid Unsupervised (RigUn), Deformable Supervised (DefSup), and Deformable Unsupervised (DefUn). From each workflow, two treatment plans – scheduled and adaptive – were generated.

Four distinct adaptive workflows were analyzed: Deformable Supervised (DefSup), Deformable Unsupervised (DefUn), Rigid Supervised (RigSup), and Rigid Unsupervised (RigUn) (Figure 1).

For the DefSup and DefUn workflows, a deformable contour propagation method was applied, wherein structures from the reference plan were deformed using system-defined algorithms to align with the anatomy on the CBCT. In contrast, for the RigSup and RigUn workflows, a rigid contour propagation method was applied, transferring structures from the reference plan directly to the CBCT without accounting for anatomical changes (Supplementary, Figure 1). In the DefSup and RigSup workflows, manual edits were performed whenever the CTV structure was found insufficient to fully cover the target, that is the affected vertebra(e). In the RigUn workflow, only translational and rotational adjustments were applied. For the DefUn workflow, no manual modifications were made to the structures.

Delineated structures from the dCT were used as a reference for contouring during treatment emulation. To ensure an unbiased approach to target recognition, the RigSup and DefSup workflows were emulated first, followed by the DefUn and RigUn workflows.

### Emulated oART treatments

The adaptive treatments were emulated using the Ethos emulator (v2.0), a virtual replica of the clinical Ethos system used in the department. The CBCTs served as the imaging and dose calculation basis for the adaptive treatments (Figure 1). All emulated treatments were conducted by an experienced adapter RTT, with each defined adaptive workflow carried out for every included treatment site.

The treatments were timed at each of the five steps: (1) anatomy review, (2) target review, (3) contour editing, (4) optimization time, and (5) plan review. The optimization time step was defined as the duration the system used to generate the two treatment plans: a scheduled plan (dose distribution from the reference plan re-calculated on the daily anatomy) and an adaptive plan (re-optimization of the dose distribution on the daily anatomy).

The total time consumption and the duration of each individual step were analyzed and compared across the different workflows.

### Segmentation comparisons

To assess the accuracy of the contouring, the CTV structure was selected as the basis for comparison, due to its clinical relevance as the primary target structure. Since the aim of the segmentation comparison was to evaluate the feasibility of different contour propagation methods, the CTV from the DefSup workflow was designated as the ‘ground truth’, against which the CTV structures generated in the DefUn and RigUn workflows were compared. Similarity between structures was evaluated using the Dice Similarity Coefficient (DSC). DSC values < 0.50, 0.50–0.59, 0.60–0.69, 0.70–0.79, and 0.80–1.00 were indicative of poor, intermediate-poor, intermediate, good-intermediate, and good contour overlap levels, respectively [16].

### Plan evaluations

To evaluate plan quality, dose metrics including CTV V97%, PTV V95%, PTV D1.00 cm³, and Spinal Cord D_max_, were extracted from the Ethos TPS and analyzed. The dose metrics from the dCT based reference plan were considered ‘ground truth’ for comparison. Differences in dose metrics between the reference plan and each of the four alternative workflows (including both adaptive and scheduled dose plans) were compared and analyzed.

### Sub-analysis of unsupervised workflow

To evaluate the potential of an unsupervised workflow, a sub-analysis was performed on the first six patients included in the study. Specifically, the goal was to assess how well the adaptive treatment plans from the DefUn workflow would have covered the manually edited structures in the DefSup workflow. For this purpose, treatment plans were generated using the contours from the DefUn workflow for optimization, and target coverage metrics (CTV V97%, PTV V95%, and PTV V90%) were evaluated using the structures from the DefSup workflow. In addition, clinical acceptability was assessed through visual inspection of isodose coverage on the DefSup contours, performed by two radiation oncologists.

### Statistical analysis

Statistical analyses were performed in R Statistical Software (v4.4.2; R Core Team, 2024) [17]. Normality was assessed using the Shapiro–Wilk test. Median values and interquartile ranges (IQR) were calculated for both overall time consumption and for each of the five adaptive workflow steps. Median and IQR were calculated for the DSC. Dosimetric differences between the reference plan and both adaptive and scheduled plans across the four workflows were expressed as median percentage differences. A Wilcoxon signed-rank test, with a significance level of 5%, was used to assess variances in both contouring and dose coverage.

## Results

### Patients included

A total of 30 patients were assessed for eligibility, with three patients excluded for not meeting the inclusion criteria. Two patients were excluded for failing to provide signed informed consent. One patient was excluded due to surgery between the dCT scan and the start of treatment, leading to significant anatomical changes between the scans. Three patients had two separate treatment sites, each planned and evaluated independently, resulting in a total of 30 targets included for emulation. Patient characteristics are summarized in Table 1.

**Table 1:** 

Patient Characteristics	No. (%) All patients (*n* = 27)
**Age**	
Median [range], years	71 [64;77]
**Sex, no. (%)**	
Men	18 (67)
Women	9 (23)
**Site of primary cancer**	
Prostate	4 (15)
Lung	4 (15)
Breast	5 (18)
Kidney	6 (22)
Other	8 (30)
**No. of treated MSCC sites**	
Single	24 (89)
Multiple	3 (11)
**No. of treated vertebrae pr. site**	
Median [range]	1 [1;2]
**Anatomical location**	
Thoracic	18 (60)
Lumbar	9 (30)
Sacral	3 (10)
**Time between dCT and MRI median [range], days**	12 [1;35]

### oART procedure time

**Figure 2:**
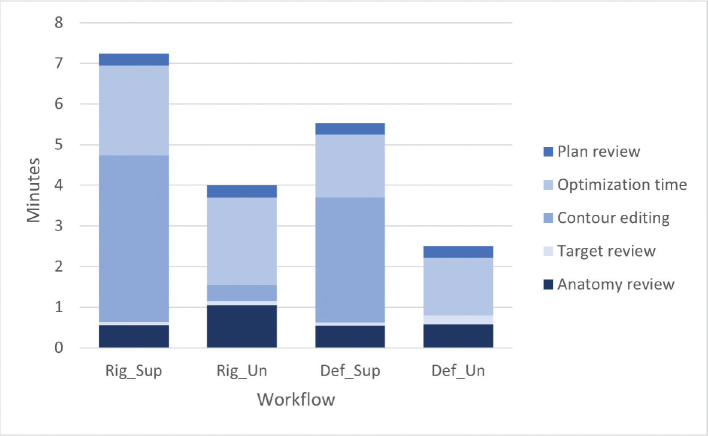
Bar chart showing the median duration of each five steps in the adaptive treatment for the Rigid Supervised (Rig_Sup), Rigid Unsupervised (RigUn), Deformable Supervised (DefSup), and Deformable Unsupervised (DefUn) workflows.

A total of 120 treatments were emulated using the Ethos emulator (Figure 2) (based on 27 patients with 30 target sites). The median [IQR] overall time for the RigSup workflow was 7.49 min [7.05; 9.10], compared to 4.53 min [4.31; 5.42] for the RigUn workflow. Similarly, the DefSup workflow had a median time of 6.57 min [6.03; 7.59], while the DefUn workflow took 3.27 min [2.25; 4.07].

### Segmentation comparisons

**Figure 3:**
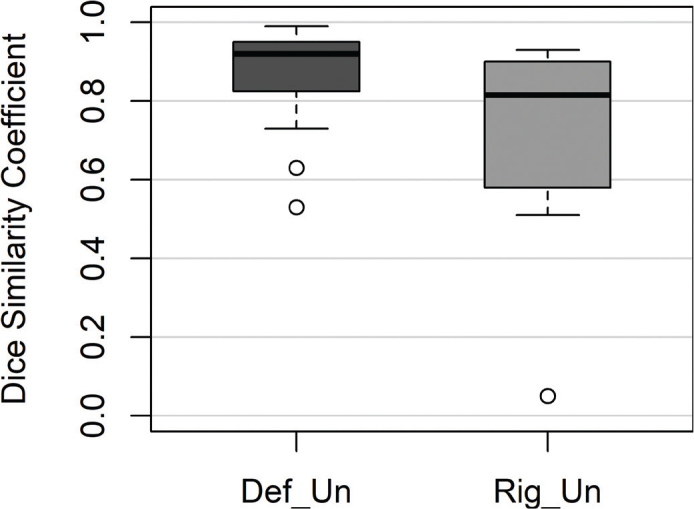
Boxplot presenting the Dice Similarity Coefficient for the Clinical Target Volume (CTV) structure from the adaptive treatment plans across all patients when comparing deformable supervised (DefSup) with deformable unsupervised (DefUn) and rigid unsupervised (RigUn).

The comparison of the CTV structure from the DefSup workflow with those from the DefUn and RigUn workflows reveals the presence of outliers in the dataset (Figure 3). A mean DSC of 0.87 ± 0.12 (good) was observed for the DefUn workflow, while the RigUn workflow showed a mean DSC of 0.66 ± 0.21 (intermediate). Despite these variations, most of the data remain within a relatively narrow range, indicating general consistency in performance in the DefUn workflow, though further investigation into the outliers may be warranted to understand their impact.

### Plan evaluations

Across all 120 treatments performed in the emulator, the adaptive dose plan was selected in every case, as it fulfilled all predefined clinical dose constraints. In contrast, none of the scheduled dose plans met all clinical goals.

When comparing the CTV V97% across the four workflows, statistically significant differences were observed for both the scheduled and adaptive plans compared to the reference plan. However, the scheduled plans showed larger median percentage differences from the reference plan, indicating a greater deviation in dose coverage compared to the adaptive plans (Supplementary, Table 2).

**Figure 4:**
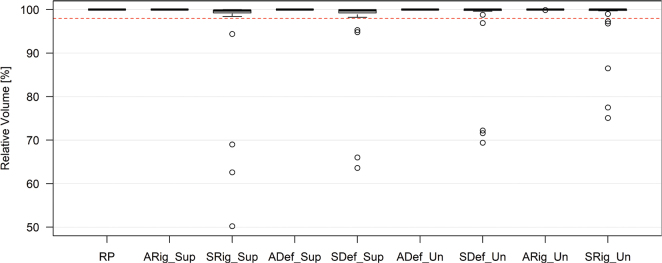
Boxplot presenting the Planning Target Volume (PTV) V95% dose from the adaptive and scheduled plans from the four defined workflows, respectively. The red dashed line indicates the clinically acceptable dose coverage of 98% for the PTV V95% goal. RP: Reference Plan, ARig_Sup: Adaptive Rigid Supervised, SRig_Sup: Scheduled Rigid Supervised, ADef_Sup: Adaptive Deformable Supervised, SDef_Sup: Scheduled Deformable Supervised, ADef_Un: Adaptive Deformable Unsupervised, SDef_Un: Scheduled Deformable Unsupervised, ARig_Un: Adaptive Rigid Unsupervised, SRig_Un: Scheduled Rigid Unsupervised.

For PTV V95%, the scheduled plans across all workflows showed statistically significant differences compared to the reference plan, indicating deviations in dose coverage. In contrast, the adaptive plans did not show significant differences, reflecting more consistent and uniform dose distribution (Supplementary Table 3). Additionally, the median dose coverage for the scheduled plans met the clinical goal for target dose, although multiple outliers in target coverage were observed (Figure 4). Regarding the PTV D1.00 cm³, both adaptive and scheduled plans showed statistically significant differences compared to the reference plan. However, only the adaptive plans consistently met the clinical constraints (Supplementary, Table 4). Finally, for the spinal cord D_max_, p-values were consistently below 0.05 for all adaptive plans, indicating a significant difference in dose distribution relative to the reference plan. In contrast, scheduled plans, regardless of the RigUn workflow, exhibited significant deviations from the clinical dose goal (Supplementary, Table 5).

### Sub-analysis of unsupervised workflow

**Figure 5:**
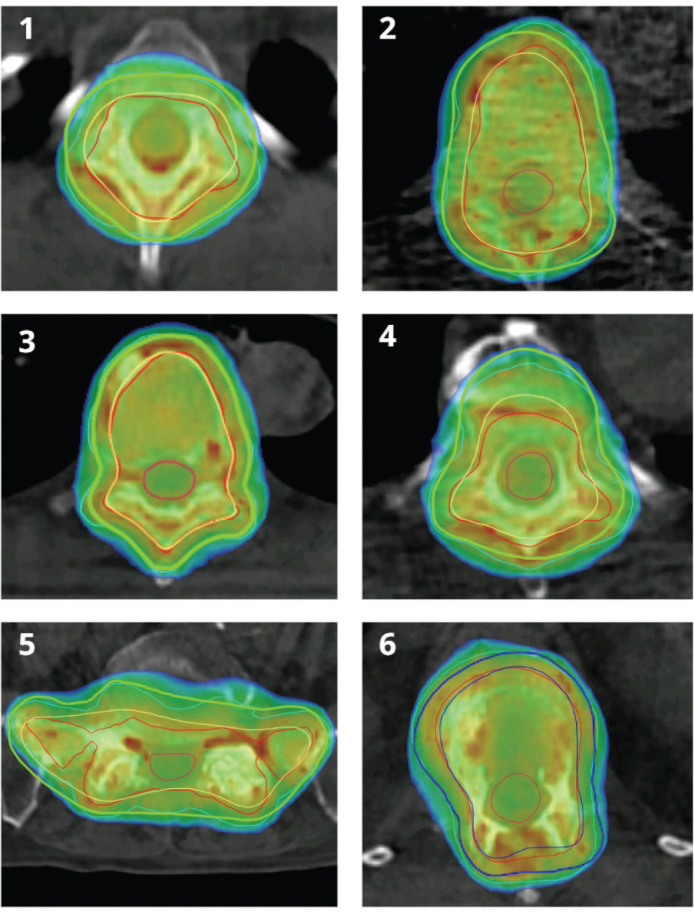
Showing isodose coverage on DefSup contours for the first six patients, after optimization of adaptive treatment plans from the DefUn. For patients 1 to 5, the supervised Planning Target Volume (PTV) is shown in bold green, the supervised Clinical Target Volume (CTV) in yellow, the unsupervised PTV in turquoise, and the unsupervised CTV in red. For patient 6, the supervised PTV is shown in bold dark blue, the supervised CTV in blue, and the unsupervised PTV and CTV in turquoise and red, respectively. The dose scale ranges from 90 to 105% of the prescribed dose.

Treatment plans created using the contours from the DefUn workflows were evaluated for target coverage using the supervised structures from the DefSup workflow. While acknowledging the inherent risks associated with an unsupervised workflow, particularly in relation to outliers, the observed results for the first six patients were not alarming (Figure 5).

Dose coverage to the PTV V95% for the first six patients was 99.0, 96.8, 99.6, 98.3, 98.8, and 99.4%. The isodose coverage for the target was assessed, and the two radiation oncologists deemed it clinically acceptable.

## Discussion and conclusion

This study demonstrates the feasibility of a simulation-free adaptive treatment workflow for patients with MSCC. The comparison of dose metrics between the dCT-based reference plan and adapted plans using different contour propagation methods showed that metrics such as CTV V97%, PTV V95%, Spinal Cord Dmax, and PTV D1.00 cm³ remained within the goal limits established during plan creation. These findings align with those of Nelissen et al. [9], who similarly demonstrated the superiority of adapted treatment plans in fulfilling clinical goals for target dose coverage while adhering to constraints for the OAR. This was further emphasized by the finding that 94.9% of the scheduled treatment plans for MSCC patients undergoing oART failed to meet at least one clinical goal.

The segmentation comparison revealed that unsupervised workflows currently demonstrate limitations, resulting in suboptimal DSC. Among the unsupervised methods, the rigid approach performed the worst compared to the deformable, though both are unsuitable for a simulation-free workflow at this time. In terms of total optimization time, the RigSup workflow proved to be the least efficient, rendering it impractical for a simulation-free oART workflow.

In terms of time consumption across the four workflows, the unsupervised workflows demonstrated superiority compared to the supervised ones, primarily due to the omitting of the contour editing step. Additionally, optimization time for the plans was reduced, as no corrections were made to the structures [9]. The overall time consumption observed in the adaptive workflows aligns with the findings of Oldenburger et al. [16], who reported a median duration of 7.50 min. Similarly, the duration of the contour editing step closely aligns with the results reported by Nelissen et al. [9], with a median duration of 3.3 min. Regarding overall time consumption, the IMRT technique was selected for this study due to its shorter optimization times compared to Volumetric Modulated Arc Therapy plans [9].

In relation to future implementation, it should be noted that a simulation-free workflow often involves the presence of a radiation oncologist or physicist [9, 11], potentially placing additional strain on clinical resources. Schuler et al. [18] highlight this as a key limitation of this approach. Nevertheless, RTT-driven oART workflows have been successfully implemented for other treatment sites [19, 20], suggesting that such an approach may also be feasible in this setting.

Moreover, the simulation-free workflow offers both clinical and patient-centered advantages. The workflow frees up pCT slots that can be allocated to other patients. It also has the potential to reduce the number of hospital visits as well as the time patients spend at the hospital, all of which are benefits that are particularly important from the patient’s perspective. This is further supported by Nelissen et al. [21], who report positive patient experiences with the simulation-free approach. For patients receiving multiple fractions, a simulation-free approach may offer reduced benefit. However, one way to increase its clinical utility in this setting could be to perform oART at the first fraction and use the CBCT from that session for the subsequent treatments. The potential of this strategy warrants further prospective evaluation.

Some limitations of this study should be acknowledged. Treatments were performed within an emulator, capturing only the time spent on specific steps within the adaptive workflow. This setup may have influenced the timing results, meaning this study does not provide complete data on the total time needed for a full oART treatment. Additionally, differences in the computation power of the emulator versus the clinical system might have influenced the time consumption. Nonetheless, in our department, a standard timeslot of 45 min is allocated for the first treatment. Therefore, although the reported results reflect only the adaptive workflow, they suggest that the treatments remain feasible within clinical practice.

Recognition bias may have influenced time consumption, as all emulations followed the RigSup, then the DefSup, potentially making the DefSup workflow faster due to familiarity. One could argue that blinding the RTT to the registration method might have mitigated the bias. However, since rigid propagation of the structures requires active involvement from the RTT during the adaptive workflow, blinding was not feasible in this context. Since all emulations were performed by a single RTT, time consumption and delineation variation might have differed if multiple RTTs had participated.

Furthermore, the analysis of the plan evaluation revealed several outliers, where the scheduled plans did not meet the target dose coverage goals. Inspection of the cases with the lowest dose coverage indicated that the issue likely arose from large anatomical variations, such as differences in patient arm positioning. Anatomical misalignment in arm positioning may have led to beam passage through the arms, thereby impacting the accuracy of target dose coverage. It should be noted that achieving clinically acceptable scheduled plans was not the focus of this study. To achieve clinically acceptable scheduled plans, or if a synthetic CT workflow is to be used, treatment plans should be generated with patient-specific beam angles that avoid regions of high anatomical uncertainty.

The DefSup workflow emerged as the most effective approach, balancing both efficiency and reliability, making it the optimal choice for simulation-free oART workflows for MSCC patients. Although the DefUn workflow holds promise for streamlining procedures, the risk of outliers currently makes them unsuitable. Nonetheless, two radiation oncologists reviewed target coverage from the treatment plans from the DefUn workflow (after optimization on the structures from the DefSup workflow) for the first six included patients and deemed them clinically acceptable. Therefore, to balance safety and efficiency, the DefSup workflow was chosen for clinical implementation, with supervision to verify the correct location of the target, and to only edit the target contour when the target is assessed to be outside of the PTV.

In conclusion, this study demonstrated the feasibility of a simulation-free oART workflow for MSCC patients, offering benefits in efficiency and patient experience regarding time spent in the department. The DefSup workflow emerged as the most optimal, paving the way for future refinements and exploration of DefUn workflows. As of late 2024, the first MSCC patient completed treatment using the DefSup workflow.

## Supplementary Material



## Data Availability

The data that support the findings of this study are available from the corresponding author upon reasonable request but are not publicly shared due to institutional policy.
