# Supplementary material for: Simulation-free cone beam CT-based online adaptive radiotherapy for metastatic spinal cord compression
Source: Acta Oncol. 2025 Aug 25;64:44040. doi: 10.2340/1651-226X.2025.44040 (PMC12398103; doi:10.2340/1651-226X.2025.44040)
Supplement: Supplementary file 1 [file AO-64-44040-s1.pdf]

Herlev Hospital

Supplementary material has been published as submitted. It has not been copyedited, or typeset by Acta Oncologica

Supplementary Materials

Table S1. Dose Constraints and Goals Implemented in Ethos TPS.

**Targets**	**Goals/Constraints**	**Acceptable Variance**
CTV	V97%≥100%	V97%≥98%
PTV	V95%≥99%	V95%≥98%
PTV	D1.00 cm3≤103%	D1.00 cm3≤105%
Spinal Cord	Dmax≤25.5 Gy	Dmax≤26.25 Gy

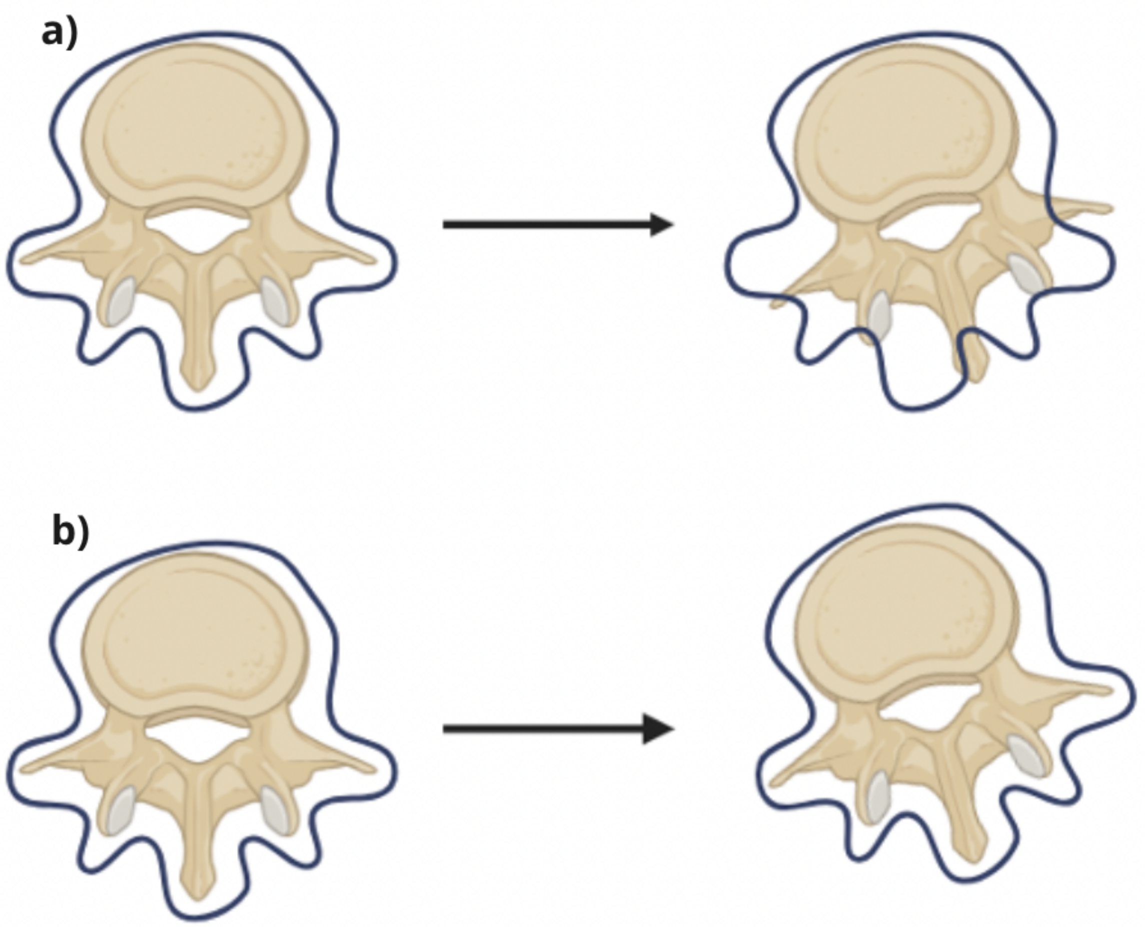

Figure S1. a) Illustration of the direct transfer of structures delineated on the reference dCT to the CBCT scan. **b)** Illustration of how the system generates and deforms the structures to match the changed anatomy on the CBCT scan.

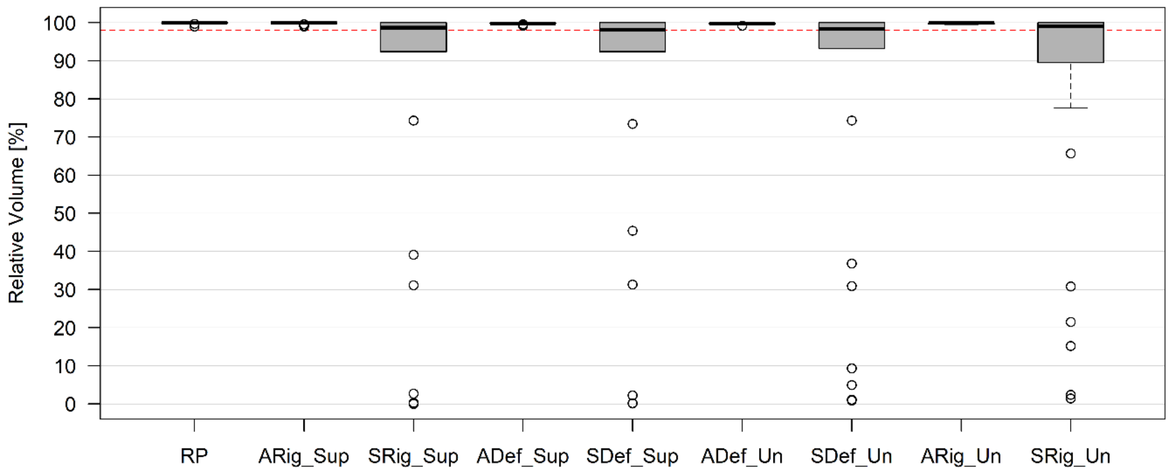

Figure S2. Boxplot presenting the CTV V97% dose from the adaptive and scheduled plans from the four defined workflows, respectively. The red dashed line indicates the clinically acceptable dose coverage of 98% for the CTV V97% goal. RP: Reference Plan, ARig_Sup: Adaptive Rigid Supervised, SRig_Sup: Scheduled Rigid Supervised, ADef_Sup: Adaptive Deformable Supervised, SDef_Sup: Scheduled Deformable Supervised, ADef_Un: Adaptive Deformable Unsupervised, SDef_Un: Scheduled Deformable Unsupervised, ARig_Un: Adaptive Rigid Unsupervised, SRig_Un: Scheduled Rigid Unsupervised.

Table S2. Median percentage differences in CTV V97% dose between the reference plan and both the scheduled and adaptive treatment plans for each of the four workflows. In addition, results from a Wilcoxon signed-rank test comparing each plan to the reference plan are presented. ARigSup: Adaptive Rigid Supervised, SRigSup: Scheduled Rigid Supervised, ADefSup: Adaptive Deformable Supervised, SDefSup: Scheduled Deformable Supervised, ADefUn: Adaptive Deformable Unsupervised, SDefUn: Scheduled Deformable Unsupervised, ARigUn: Adaptive Rigid Unsupervised, SRigUn: Scheduled Rigid Unsupervised.

**Plan**	**Median Difference (%)**	**p-value**
ARigSup	-0.11	<0.05
SRigSup	-1.26	<0.05
ADefSup	-0.11	<0.05
SDefSup	-1.81	<0.05
ADefUn	-0.11	<0.05
SDefUn	-1.51	<0.05
ARigUn	-0.11	<0.05
SRigUn	-0.51	<0.05

Table S3. Median percentage differences in PTV V95% dose between the reference plan and both the scheduled and adaptive treatment plans for each of the four workflows. In addition, results from a Wilcoxon signed-rank test comparing each plan to the reference plan are presented.
ARigSup: Adaptive Rigid Supervised, SRigSup: Scheduled Rigid Supervised, ADefSup: Adaptive Deformable Supervised, SDefSup: Scheduled Deformable Supervised, ADefUn: Adaptive Deformable Unsupervised, SDefUn: Scheduled Deformable Unsupervised, ARigUn: Adaptive Rigid Unsupervised, SRigUn: Scheduled Rigid Unsupervised.

**Plan**	**Median Difference (%)**	**p-value**
ARigSup	0.00	1
SRigSup	-0.20	<0.05
ADefSup	0.00	1
SDefSup	-0.15	<0.05
ADefUn	0.00	1
SDefUn	0.00	<0.05
ARigUn	0.00	1
SRigUn	0.00	<0.05

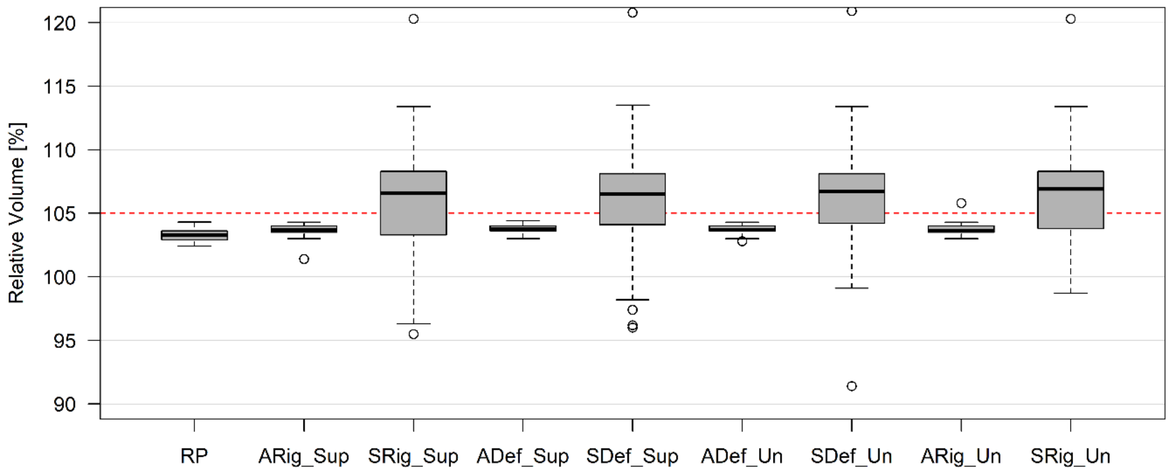

Figure S3. Boxplot presenting the PTV D1.00 cm^3 % dose from the adaptive and scheduled plans from the four defined workflows, respectively. The red dashed line indicates the clinically acceptable dose coverage of 105% for the PTV D1.00 cm^3 % goal. RP: Reference Plan, ARig_Sup: Adaptive Rigid Supervised, SRig_Sup: Scheduled Rigid Supervised, ADef_Sup: Adaptive Deformable Supervised, SDef_Sup: Scheduled Deformable Supervised, ADef_Un: Adaptive Deformable Unsupervised, SDef_Un: Scheduled Deformable Unsupervised, ARig_Un: Adaptive Rigid Unsupervised, SRig_Un: Scheduled Rigid Unsupervised.

Table S4. Median percentage differences in PTV D1.00 cm^3 % dose dose between the reference plan and both the scheduled and adaptive treatment plans for each of the four workflows. In addition, results from a Wilcoxon signed-rank test comparing each plan to the reference plan are presented.
ARigSup: Adaptive Rigid Supervised, SRigSup: Scheduled Rigid Supervised, ADefSup: Adaptive Deformable Supervised, SDefSup: Scheduled Deformable Supervised, ADefUn: Adaptive Deformable Unsupervised, SDefUn: Scheduled Deformable Unsupervised, ARigUn: Adaptive Rigid Unsupervised, SRigUn: Scheduled Rigid Unsupervised.

**Plan**	**Median Difference (%)**	**p-value**
ARigSup	0.43	<0.05
SRigSup	3.59	<0.05
ADefSup	0.47	<0.05
SDefSup	3.49	<0.05
ADefUn	0.57	<0.05
SDefUn	3.63	<0.05
ARigUn	0.38	<0.05
SRigUn	3.97	<0.05

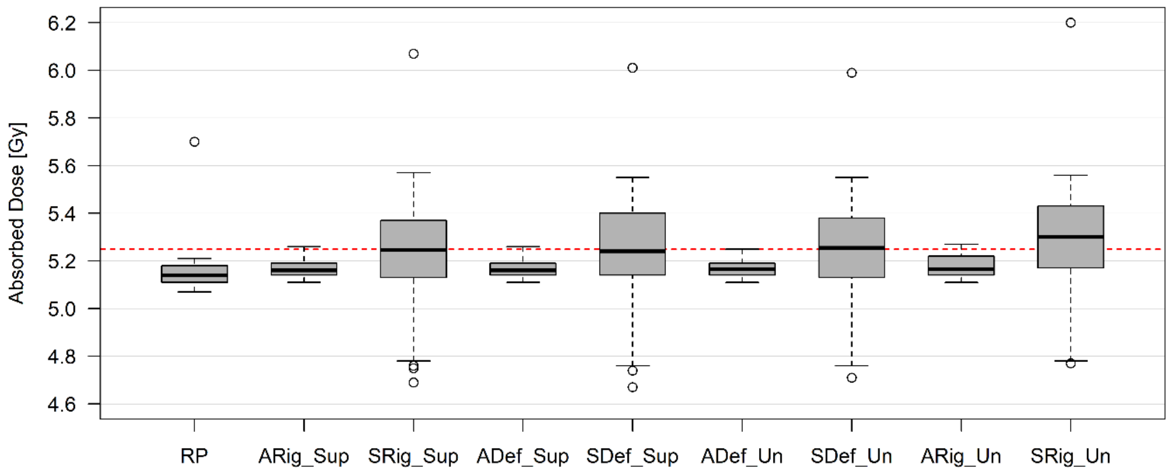

Figure S4. Boxplot presenting the Spinal Cord D_{Max} dose from the adaptive and scheduled plans from the four defined workflows, respectively. The red dashed line indicates the clinically acceptable dose coverage of 5.25 Gy for the Spinal Cord D_{Max} goal. RP: Reference Plan, ARig_Sup: Adaptive Rigid Supervised, SRig_Sup: Scheduled Rigid Supervised, ADef_Sup: Adaptive Deformable Supervised, SDef_Sup: Scheduled Deformable Supervised, ADef_Un: Adaptive Deformable Unsupervised, SDef_Un: Scheduled Deformable Unsupervised, ARig_Un: Adaptive Rigid Unsupervised, SRig_Un: Scheduled Rigid Unsupervised.

Table S5. Median percentage differences in Spinal Cord D_{Max} dose dose between the reference plan and both the scheduled and adaptive treatment plans for each of the four workflows. In addition, results from a Wilcoxon signed-rank test comparing each plan to the reference plan are presented.
ARigSup: Adaptive Rigid Supervised, SRigSup: Scheduled Rigid Supervised, ADefSup: Adaptive Deformable Supervised, SDefSup: Scheduled Deformable Supervised, ADefUn: Adaptive Deformable Unsupervised, SDefUn: Scheduled Deformable Unsupervised, ARigUn: Adaptive Rigid Unsupervised, SRigUn: Scheduled Rigid Unsupervised.

**Plan**	**Median Difference (%)**	**p-value**
ARigSup	0.39	<0.05
SRigSup	1.95	0.11
ADefSup	0.19	<0.05
SDefSup	2.05	0.09
ADefUn	0.39	<0.05
SDefUn	2.34	0.13
ARigUn	0.58	<0.05
SRigUn	3.31	<0.05

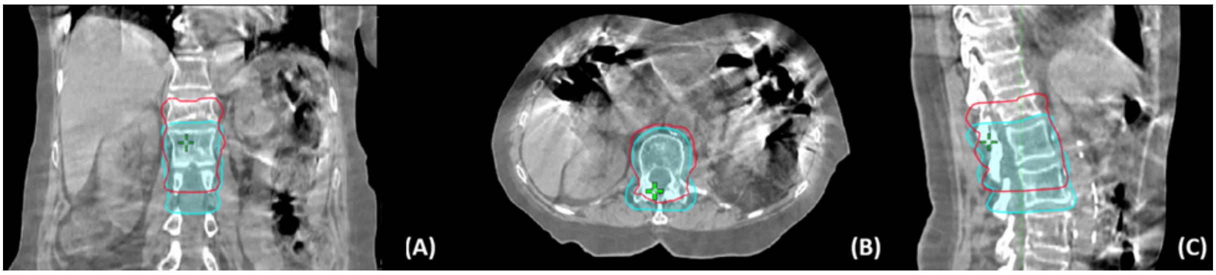

Figure S5. PTV contours of an outlier (95% HD=18.02 mm and DSC=0.63) for the segmentation comparison. PTV from the adaptive deformable supervised workflow (in red) vs PTV from the adaptive deformable unsupervised workflow (in light blue) for A) frontal view B) transversal view and C) sagittal view.
